# Polyomavirus Associated Nephropathy: Frequency and Graft Survival Analysis in Northeast of Iran

**DOI:** 10.30699/IJP.2021.128489.2403

**Published:** 2021-03-02

**Authors:** Shirin Taraz Jamshidi, Khadijeh Sajjadian, Maryam Emadzadeh, Malihe Saber Afsharian, Mahmoud Reza Kalantari, Anita Alenabi, Abbas Ali Zeraati, Ali Emadzadeh

**Affiliations:** 1 *Kidney Transplantation Complications Research Center, Mashhad University of Medical Sciences, Mashhad. Iran*; 2 *Student Research Committee, Mashhad University of Medical Sciences, Mashhad, Iran*; 3 *Clinical Research Unit, Mashhad University of Medical Sciences, Mashhad, Iran*; 4 *Department of Pathology, Faculty of Medicine, Mashhad Azad University, Mashhad, Iran*; 5 *Cancer Molecular Pathology Research Center, Mashhad University of Medical Sciences, Mashhad, Iran*

**Keywords:** Frequency Graft survival analysis Polyomavirus-associated nephropathy

## Abstract

**Background & Objective:**

Polyomavirus-associated nephropathy (PVAN), mainly caused by the BK virus, is one of the most important infectious complications of kidney transplantation. The leading histopathologic characteristics of PVAN is viral cytopathic effects, such as nucleomegaly with smudged or clumped chromatin and intranuclear ground-glass inclusion, mostly in tubular epithelial cells. Moreover, tubular necrosis, tubulitis, interstitial inflammation, atrophy, and fibrosis have been noted. Positive immunohistochemistry (IHC) staining for SV-40 highlights the infected epithelial cells of renal tubules.

**Methods:**

A total of 85 core needle biopsies of transplanted kidneys were evaluated histologically and were stained for SV-40 using the IHC method. In addition, a follow-up of graft failure was performed.

**Results:**

Our findings revealed that the frequency of polyomavirus infection in kidney transplant patients in the Northeast of Iran is 4.7%. There was no significant correlation between PVAN and graft rejection. Although a higher rate of graft loss was observed in PVAN patients, in comparison with non-PVAN patients (25% vs. 14.8%), the difference was not statistically significant. Moreover, patients with immunohistochemically confirmed PVAN and those with histopathologic features of viral-like cytopathic effects had significantly lower graft survival in the follow-up period (42.5 vs. 196.8 months and 109.4 vs. 205.7 months, respectively).

**Conclusion:**

The frequency of polyomavirus infection in kidney transplant patients in the Northeast of Iran is 4.7%. There was no significant correlation between PVAN and graft rejection. Furthermore, we observed that polyomavirus infection accelerates the course of graft loss.

## Introduction

Human polyomaviruses are the members of the polyomaviridae family, which includes ubiquitous viruses, such as the BK virus and JC virus. These agents cause asymptomatic infections among immunocompetent people. However, immunosuppression can lead to the activation of these viruses. Polyomavirus-associated nephropathy(PVAN), mainly caused by the BK virus, is one of the most important infectious complications of kidney allograft (1). In the majority of patients, a biopsy of allograft kidney is taken in case of unjustified clinical presentation, which could be due to the BK virus. Overt polyomavirus nephropathy may emerge as an incidental finding in periodical renal biopsies in patients with stable disease (2-4). One of the main histopathological characteristics of PVAN is viral cytopathic effects, including nucleomegaly, smudged or clumped chromatin, and intranuclear ground-glass inclusion with or without a halo mostly in the epithelium of tubules. Other features of PVAN include tubular necrosis, tubulitis, interstitial inflammation, atrophy, and fibrosis. However, allograft renal biopsies are performed with different clinical indications, which may result in various histomorphological findings in PVAN. Positive immunohistochemistry (IHC) staining for SV-40 highlights the infected epithelial cells of renal tubules (5, 6). In different countries, the incidence of histologically confirmed polyomavirus nephropathy is reported to be approximately 1%-10% with a large diversity based on regions and institutes (7, 8). This study aimed to assess the frequency of PVAN in the allograft kidney biopsies and its correlation with histopathologic findings and graft survival in the Northeast of Iran.

## Materials and Methods

In the current study, core needle biopsies of transplanted kidneys were obtained from the archives of the pathology departments of three major medical centers of Ghaem Educational Hospital, Imam Reza Educational Hospital, and Mashhad Pathobiology Laboratory, Mashhad, Iran during 2009-2019. Eighty-five cases with complete clinical data and sufficient tissue in paraffin blocks were included in the study. The specimens devoid of the medullary portion were excluded. All hematoxylin and eosin-stained slides were reviewed by two independent pathologists. Histopathologic data, such as nuclear changes imply viral-like cytopathic effects, namely nucleomegaly, smudged/clumped chromatin, and intranuclear ground-glass inclusion with or without a halo in the epithelium of renal tubules. The histopathologic evidence of graft rejection (classified according to Banff classification 2018), histopathologic features of recurrent or de novo glomerulopathies, and calcineurin inhibitor nephrotoxicity features were recorded. Demographic and clinical data, including age, gender, and serum creatinine level at the time of biopsy were obtained from medical records. In the follow-up, the detection of any graft failure was defined as the loss of graft function and permanent need for dialysis any time after the transplantation. The IHC was performed on formalin-fixed paraffin-embedded tissue sections using the SV40 antibody (Calbbiochem, clone PAb416, USA, dilution 1:300) to detect large T-antigen of a polyomavirus in infected cells. The slides were evaluated by two pathologists and the results were considered positive in the case of at least one cell with nuclear staining of SV40 antibody.

## Results

**Figure 1:**
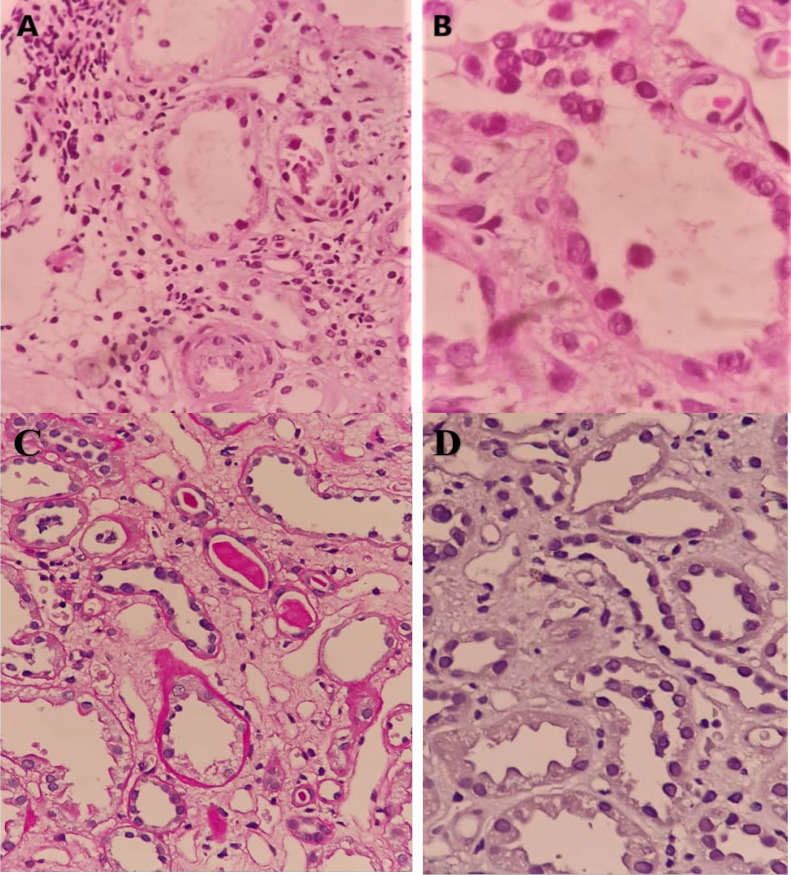
Sections of medullary regions stained by Hematoxylin and Eosin show atrophic tubules lined by epithelial cells with nuclear enlargement and coarse chromatin in an SV40-positive case (A); High power reveals intranuclear inclusions in the same case (B); Nucleomegaly and smudged chromatin in tubular epithelial cells that interpreted as a viral-like cytopathic effect by periodic acid-Schiff stain (C); The same case as panel C with negative IHC result ( SV40 immunostain) (D)

**Figure 2:**
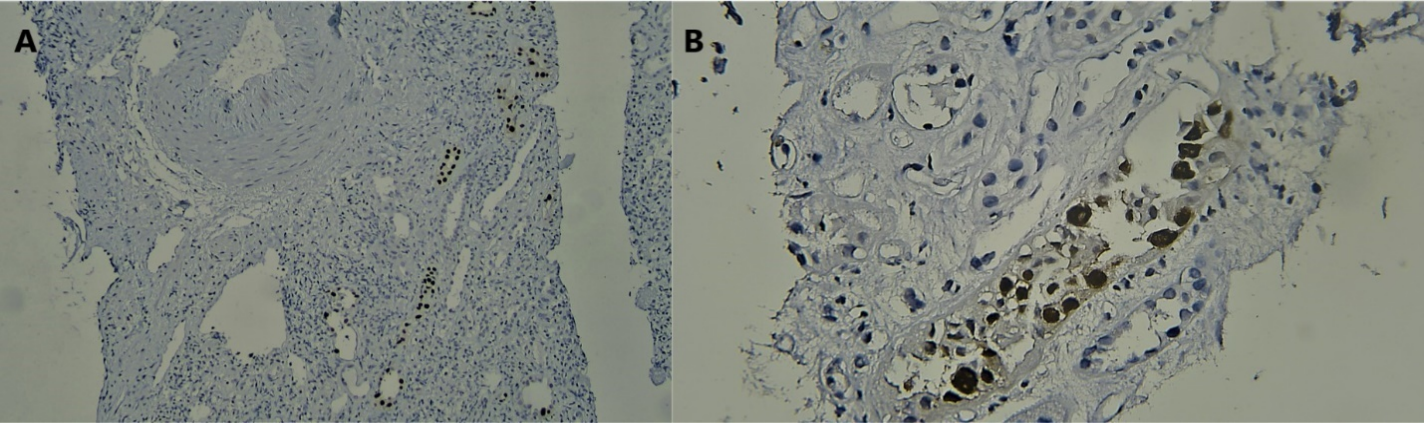
SV40 immunostain is positive in tubular epithelial cells; Low power (A) and high power views (B)

In the present investigation, 85 needle biopsies of transplanted kidneys were evaluated. Most patients were male (N=50, 58.8%) and the mean age of the patients was 35.87±13 years. In histopathologic evaluation, viral-like cytopathic changes were observed in 18 (21.2%) biopsies (Figure 1), however, only four (4.7%) of the cases were SV40-positive (Figure 2). Therefore, only one-fourth of the viral-like cytopathic features could be attributed to the polyomavirus infection. In the histopathologic evaluation, the evidence of graft rejection (based on Banff classification system, 2018) was observed in 68 (80%) biopsies as T cell-mediated rejection (TCMR) in 49 (72%) patients, antibody-mediated rejection (AMR) in six (8.9%) patients confirmed by C4d positivity in IHC, and combined TCMR and AMR in 13 (19.1%) cases. Based on the medical records of patients the results of polymerase chain reaction (PCR) for detecting plasma BK virus load were available in 62 (out of 85) patients. A BK virus replication of more than 10000 copy/mL was considered as threshold for positive results. BK virus genome was amplified in four cases, which were also positive for SV40. Immunohistochemically negative cases had negative PCR results regardless of their histopathologic findings. Consequently, the agreement between PCR and IHC was 100% (kappa=1, *P*<0.0001). Additional histopathological findings are listed in Table 1. The mean age of the polyomavirus-positive patients, including three men and one woman was 40±13.11 years. The mean serum creatinine level at biopsy time was 2.47±1.08 mg/dL in this group. In comparison, the mean age of polyomavirus-negative patients, including 47 men and 34 women was 35.66±13.07 years. The mean serum creatinine level at biopsy time in this group was found as 2.96±2.02 mg/dL. The difference between these values was not statistically significant, which might be due to limited sample size. 

**Figure 3:**
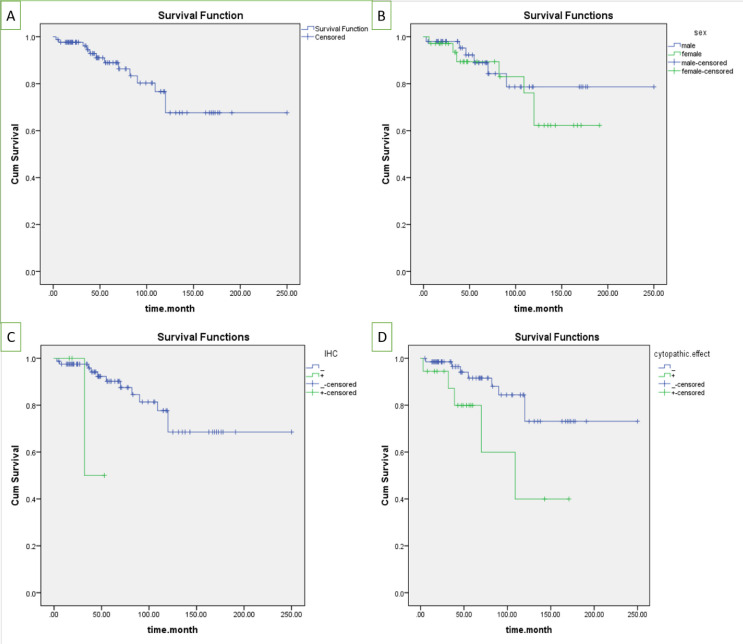
**A) **The mean graft survival in all patients was 194.6 months (95% CI: 168.2-221); B) The mean graft survival was 151 months (95% CI: 126.4-175.6) in women and 209.2 months (95% CI: 179.1-239.3) in men (*P*=0.554); C) The mean graft survival in polyomavirus-infected kidneys was significantly lower than non-infected ones (24.5 months 95% CI: 27.9-57 and 196.8 months 95% CI: 170.4-223.3, *P*=0.049); D) The mean graft survival in samples with features suggestive of viral cytopathic effects was significantly lower than others (109.41 months 95% CI: 69.5-149.2 and 205.75 months 95% CI: 178-223.4, *P*=0.027)

In the survival analysis, the starting point was transplantation time and graft failure was considered as the final event. The minimum length of follow-up was three months and the maximum length was 250 months. During the follow-up period, 13 (15.3%) patients lost their graft function and permanently needed dialysis. Out of four SV40-positive patients, only 1 (25%) showed graft failure during this period. This case had BK nephropathy class B according to the Banff Working Group classification (9). From the remaining three SV-40 positive patients, two were classified in class B and one was class A. There was no statistically significant difference in BK nephropathy stage/class between patients with and without graft loss. This was probably due to the small sample size. Graft survival mean was 194.6 months (95% CI: 168.2- 221) (Figure 3a) with 151 (95% CI 126.4-175.6) and 209.2 months (95% CI 179.1-239.3) in women and men, respectively (*P*=0.554) (Figure 3b). The mean graft survival in polyomavirus-infected kidneys was significantly shorter than non-infected ones (24.5 months, 95% CI: 27.9-57 vs. 196.8 months, 95% CI: 170.4-223.3, *P*=0.049) (Figure 3c). Moreover, the mean graft survival in biopsies with viral-like cytopathic effects was significantly lower than others (109.41 months 95% CI: 69.5-149.2 vs. 205.75 months 95% CI: 178-223.4, *P*=0.027) (Figure 3d). It should be mentioned that the histologic features of viral infections, such as nucleomegaly and homogenized chromatin are not specific to polyomavirus and could be found in cytomegalovirus infection or reactive conditions. 

**Table 1:** Histopathological findings of the core biopsies of transplanted kidneys

Histopathological findings	Frequency (%)
Glomerulopathies	
Ig A nephropathy	**3 (3.5)**
Diabetic nephropathy	**2 (2.4)**
Hemolytic uremic syndrome (HUS)	**4 (4.7)**
Focal segmental glomerulosclerosis (FSGS)	**11 (12.9)**
Membranoproliferative glomerulonephritis (MPGN)	**5 (5.9)**
Chronic calcineurin inhibitor nephrotoxicity (CIN)	**24 (28.2)**
Mild	**19**
Moderate	**5**
Severe	**0**
Interstitial fibrosis and tubular atrophy (IFTA)	**53 (62.4)**
Mild	**32**
Moderate	**21**
Severe	**0**

## Discussion

Polyomavirus infection has a high frequency in the general population as seropositivity for BK virus and JC virus in different populations and various age groups reaches up to 99% and 78%, respectively (10-13). Although infection with this virus in healthy people remains latent, the BK virus reactivates in immunocompromised patients, such as the recipients of solid-organ transplants who receive immunosuppressive agents (14). Reactivation of the BK virus causes BK virus nephropathy in 1%-10% of kidney allograft recipients. 

Hirsch* et al.* reviewed 11 studies showing the mean prevalence of 4.91% for BK nephropathy, which is almost similar to our findings (7, 8). In the study carried out by Ramos *et al.*, the incidence of PVAN was 5.1 (15). In our study on 85 patients with an allograft kidney transplant, PVAN was confirmed in four (4.7%) cases by the IHC method. However, Safari *et al.* reported a higher frequency of 13.1% in the Iranian population (16). Among the studies performed in Iran, Eidgahi *et al.* studied 247 renal transplant patients in the Northeast of Iran and found only two cases with positive PCR results for BK virus in the urine. Although their reported frequency is much lower than ours, the methodology is different and histopathological evaluation was not performed in their study (17). 

Graft loss following PVAN is estimated as 10%-80% in different centers (7). In the present study, only one case of PVAN (25%) had graft failure. In the non-PVAN group, graft failure was detected in 12 of 81 patients (14.8%). Therefore, the rate of graft failure was higher in the PVAN group. However, the difference was not statistically significant, which might be due to the small sample size. On the other hand, we failed to show any statistical relationship between PVAN and the graft rejection rate. There is no consensus on the role of polyomavirus infection in increasing the chance of transplant rejection. Cheungpasitporn *et al.* revealed that BK nephropathy is a risk factor for donor-specific antibody formation which in turn results in antibody-mediated graft rejection (18). Based on graft survival analysis, we concluded that graft survival significantly diminishes in the presence of either polyomavirus infection (confirmed by IHC) or histopathologic evidence of a viral cytopathic effect. Schwarz and colleagues reported 14% graft loss in BK virus nephropathy patients along with lower renal function, in comparison with BK virus-negative cases. Most of the patients in the latter investigation represented class B histology similar to our PVAN patients (19). In histopathologic evaluation, we noticed some features of viral-like cytopathic effects in 21.2% of biopsies, while only 4.7% turned out to be positive for SV40. This could be explained by reactive nuclear changes secondary to chronic inflammation mimicking nuclear changes in viral infections or cytopathic effects due to other viral infections, such as cytomegalovirus (20).

The current study indicated a complete agreement between the PCR method for the evaluation of BK virus plasma viral load and SV40 IHC staining in paraffin blocks. This study was conducted for the first time in the Northeast of Iran using the IHC method for renal biopsies to confirm PVAN in transplanted kidneys and analyze graft survival. The limitation of this retrospective study was the low sample size, which was inevitable due to the insufficient tissue material and incomplete medical records.

## Conclusion

In conclusion, the results of the present study revealed that the frequency of polyomavirus infection in kidney transplant patients in the Northeast of Iran is 4.7%. Patients with PVAN and those with viral-like cytopathic effects had significantly lower graft survival in the subsequent period. Consequently, we conclude that polyomavirus infection accelerates graft loss.
